# Effect of Dual-Task Conditions on Gait Performance during Timed Up and Go Test in Children with Traumatic Brain Injury

**DOI:** 10.1155/2018/2071726

**Published:** 2018-10-04

**Authors:** Rabiatul Adawiah Abdul Rahman, Fazira Rafi, Fazah Akhtar Hanapiah, Azlina Wati Nikmat, Nor Azira Ismail, Haidzir Manaf

**Affiliations:** ^1^Centre of Physiotherapy, Faculty of Health Sciences, Universiti Teknologi MARA, Puncak Alam Campus, 42300 Puncak Alam, Selangor, Malaysia; ^2^Department of Rehabilitation Medicine, Faculty of Medicine, Universiti Teknologi MARA, Sungai Buloh Campus, 47000 Sungai Buloh, Selangor, Malaysia; ^3^Clinical and Rehabilitation Exercise Research Group, Faculty of Health Sciences, Universiti Teknologi MARA, Puncak Alam Campus, 42300 Puncak Alam, Selangor, Malaysia; ^4^Department of Psychiatry, Faculty of Medicine, Universiti Teknologi MARA, Sungai Buloh Campus, 47000 Sungai Buloh, Selangor, Malaysia; ^5^Department of Rehabilitation Medicine, Hospital Sungai Buloh, Jalan Hospital, 47000 Sungai Buloh, Selangor, Malaysia

## Abstract

**Background:**

Tasks requiring simultaneous mobility and cognition (dual tasks) have been associated with incidence of falls. Although these deficits have been documented in individuals with neurologic disorder, the effect of dual task in children with traumatic brain injury has not been fully explored.

**Objective:**

To investigate the effect of dual-task (dual-motor and dual-cognitive task) conditions on spatiotemporal gait parameters during timed up and go test in children with traumatic brain injury.

**Methods and Material:**

A total of 14 children with traumatic brain injury and 21 typically developing children participated in this case-control study. Functional balance was assessed before the actual testing to predict the risk of falls. Timed up and go test was performed under single-task and dual-task (dual-motor and dual-cognitive task) conditions. Spatiotemporal gait parameters were determined using the APDM Mobility Lab system. The descriptive statistics and* t*-test were used to analyze demographic characteristics and repeated measure ANOVA test was used to analyze the gait parameters.

**Results:**

Under dual-task (dual-motor and dual-cognitive task) conditions during the timed up and go test, gait performance significantly deteriorated. Furthermore, the total time to complete the timed up and go test, stride velocity, cadence, and step time during turning were significantly different between children with traumatic brain injury and typically developing children.

**Conclusions:**

These findings suggest that gait parameters were compromised under dual-task conditions in children with traumatic brain injury. Dual-task conditions may become a component of gait training to ensure a complete and comprehensive rehabilitation program.

## 1. Introduction

Traumatic brain injury (TBI) is one of the most common causes of disability among children worldwide with an estimation of 3 million children experiencing TBI yearly [1]. TBI may cause long-term limitations in mobility and activities such as walking depending on the severity of the impairments. Characteristics of gait abnormalities among children with TBI include slower gait speed, decreased cadence, shorter stride lengths, and increased gait variability [2, 3]. In addition, step length variability has been reported to be consistently higher in those with TBI than in healthy controls, possibly because of the greater challenges in maintaining dynamic stability during gait, especially when performing more challenging tasks [4]. Increased step length variability decreases balance performance in children with TBI [2, 3]. Thus, recovery towards independent and safe walking is essential for children with TBI to ensure that they become independent at home and in the community.

Walking is a primary daily activity in everyday life. It is not a spontaneous process as it is often accompanied by numerous types of concurrent cognitive and motor tasks [5, 6]. Therefore, a certain level of attention is required while walking [5]. Impaired attention is also commonly observed in children with TBI and is a factor that may affect gait [5]. This possibly occurs because the gait is less automatic and more dependent on central cognitive processing; therefore, the concurrent performance of two challenging attentional tasks overrides available resources [7, 8]. Another explanation could be the cognitive impairment associated with the inability to properly distribute or allocate attention, such as divided attention or sustained attention, as appropriate to the type of task and surroundings [9]. As attention is impaired in children with TBI, they may become overloaded with competing attention demands, leading to their decreased performance in one or all tasks [10].

The timed up and go (TUG) test is a valid and reliable assessment tool to examine mobility and balance performance in multiple populations. Previous study showed TUG test has demonstrated good within-session reliability among children with TBI [11]. It measures the total time taken to complete its major components: sit-to-stand, straight walking, turning, and turn-to-sit [11–13]. Moreover, the TUG test comprises dual-task conditions, which require individuals to simultaneously perform more than one task. As the walking task requires high attention demand, dual-task conditions (both motor and cognitive tasks) often impair walking and balance performance. The combination of motor and cognitive disabilities in individuals with TBI increases their risk of falls which can result in recurrent head injuries [14]. Previous studies have shown that dual-task conditions increased the time taken to complete tasks during the TUG test, with a greater effect in stroke survivors [15, 16]. The results of these studies suggest that the dual-task TUG test further challenges gait stability and, thus, may provide a more comprehensive assessment of balance capacity.

Therefore, this study aimed to examine the effect of dual-task conditions on gait performance during the modified TUG test in children with TBI and age-matched typically developing (TD) children. We hypothesized that children with TBI would present with greater deterioration in gait performance during the modified TUG test, especially under dual-task conditions, than TD children.

## 2. Materials and Methods

### 2.1. Participants

A total of 14 children with TBI (12 boys and 2 girls) and 21 TD children (17 boys and 4 girls) participated in this case-control study. The determination of sample size of the participants in this study was calculated using the G-Power 3 software. The following parameters were set: effect size (0.25), alpha (0.05), 1-beta (0.9), groups (2), measures (3), and Critical F (3.13) which resulted in total sample size of 36 participants with actual power 0.9. The mean age, height, and weight were 11.6 ± 2 years, 141 ± 17 cm, and 40.4 ± 20.5 kg in children with TBI and 11.4 ± 2.3 years, 142 ± 15 cm, and 41.2 ± 17.2 kg in TD children. No significant differences were found between the groups. Children with TBI were recruited from a government-funded hospital through purposive sampling, whereas TD children were recruited from local primary and secondary schools and matched to the children with TBI by age. Inclusion criteria for the children with TBI were as follows: (1) post-TBI duration of at least 6 months, (2) age ranging between 8 and 14 years, (3) Glasgow Coma Scale (GCS) score ≤12 at admission as determined by a physician, (4) ability to walk >10 m independently without walking aid and physical assistance, (5) ability to hold a tray, (6) ability to perform simple arithmetic calculations, and (7) demonstrating understanding of the purpose of the study. Participants with visual field deficits based on results of confrontation tests and those who underwent orthopedic surgeries in the past 6 months were excluded from the study. The study protocol was approved by the institutional research ethics committee (NMRR ID: NMRR-15-2321-28730), and parents/guardians of all participants signed an informed consent form for the children's participation in the study.

### 2.2. Instrumentation

Spatiotemporal gait parameters were determined using the APDM Mobility Lab™ system (Mobility Lab, APDM Inc., Portland, OR) based on previously validated algorithms [17, 18]. The APDM Mobility Lab™ system is a valid and reliable measurement tool to evaluate spatiotemporal gait parameters in neurological conditions [19]. This study used three Opal inertial measurement units (IMU; APDM Inc., Portland, OR) for each participant. These three Opal sensors were positioned on the posterior trunk at L5 and both shanks of the participants using adjustable Velcro straps to detect basic gait events. These IMUs have been proven to have a moderate to excellent test-retest reliability (0.56 < intraclass correlation coefficient < 0.82) [19]. All the sensors were configured for synchronized recording and real-time data acquisition at a sampling rate of 128 Hz, and data from these sensors were wirelessly streamed to a laptop. We also assessed the functional balance of participants using the Pediatric Balance Scale (PBS), which was adapted from the Berg Balance Scale, to predict the risk of falls. The PBS is a 5-point scale that evaluates 14 tasks similar to functional daily activities in children; its maximum total score is 56 [20].

### 2.3. Testing Procedure

**Figure 1:**
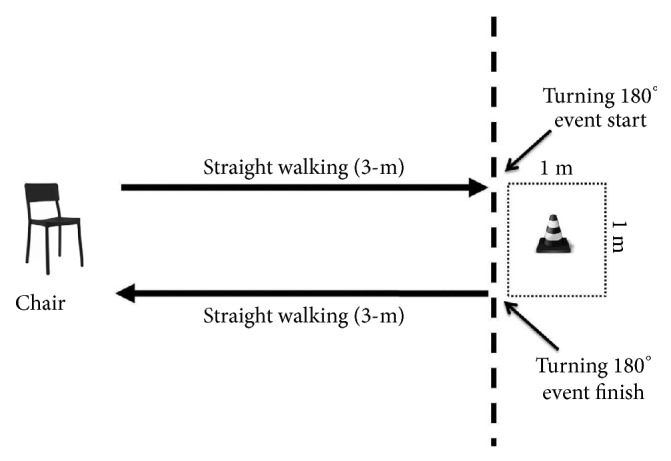
Layout for the modified timed up and go test.

The demographic and clinical data were collected after informed consent was provided. Upon the completion of the clinical assessment, the participants were instructed to perform the modified TUG test following the modifications for children described in the study by Williams et al [12]. The testing procedure was conducted in a gymnasium or school hall with standard hard and even floor surface. The layout for the modified TUG test was marked on the floor (3-m and turning areas), and these marks were clearly shown to the participants (Figure 1). The participants wore their regular footwear during the testing procedure. We demonstrated the procedure for each task. The participants were given one practice trial so that they became familiar with the test and understood it before executing the real trial. Each task was executed three times with 5-min rest provided between each trial to prevent fatigue effect on the participant's performance.

Under the single-task condition, participants were instructed to rise from a standard armless chair, walk in a straight line at their normal comfortable pace for 3-m, turn 180°, return to the chair, and sit down. Under the dual-motor task condition, participants performed the modified TUG test while holding an empty tray with both hands. Under the dual-cognitive task condition, participants performed the modified TUG test while verbally counting backwards by 1 consecutively from a given number (any number from 20 to 100). For example, if the given number was 10, participants responded “9, 8, 7,” etc. Participants were instructed to focus on the counting backwards task by counting as accurately and as fast as they could. Trials were considered failed and repeated when participants committed errors on the counting backwards task. The order of the single and dual tasks was randomized to minimize the effects of learning and fatigue.

### 2.4. Statistical Analysis

We performed an automated movement analysis to identify the straight walking and turning components of the modified TUG test and reported the following measures: (1) total time to complete the modified TUG test; (2) straight walking phase (stride velocity, stride length, cadence, gait cycle time, double support [%], swing, and stance [%]); and (3) turning phase (turning duration, step time, and step time before turning). The analysis was conducted to examine whether the effect of dual-task conditions on each phase of the modified TUG test is similar or different.

Data were analyzed using IBM SPSS Statistics version 21.0 (SPSS Inc., Chicago, IL). Data cleaning was performed to detect any missing values or outliers. Descriptive statistics were calculated, and tests for normality were conducted for all outcome variables. An independent* t*-test was used to compare the demographic data between children with TBI and TD children. Repeated-measures analysis of variance with single-task and dual-task (dual-motor and dual-cognitive task) conditions as the within-subject factor and the group (children with TBI vs. TD children) as the between-subject factor was performed to analyze the gait parameters. Post hoc Bonferroni comparisons were performed whenever the repeated-measures analysis of variance revealed a significant difference (p < 0.05).

## 3. Results

### 3.1. Participants

The characteristics of participants are presented in Table 1. The average GCS score for children with TBI was 9 ± 2 and time away from event was 2.4 ± 1.5 years. There was no statistical difference in age, body weight, and body height between both groups. In contrast, a significant difference in PBS score (p < 0.034) between both groups was found, with children with TBI having a lower mean PBS score.

### 3.2. TUG Time

**Figure 2:**
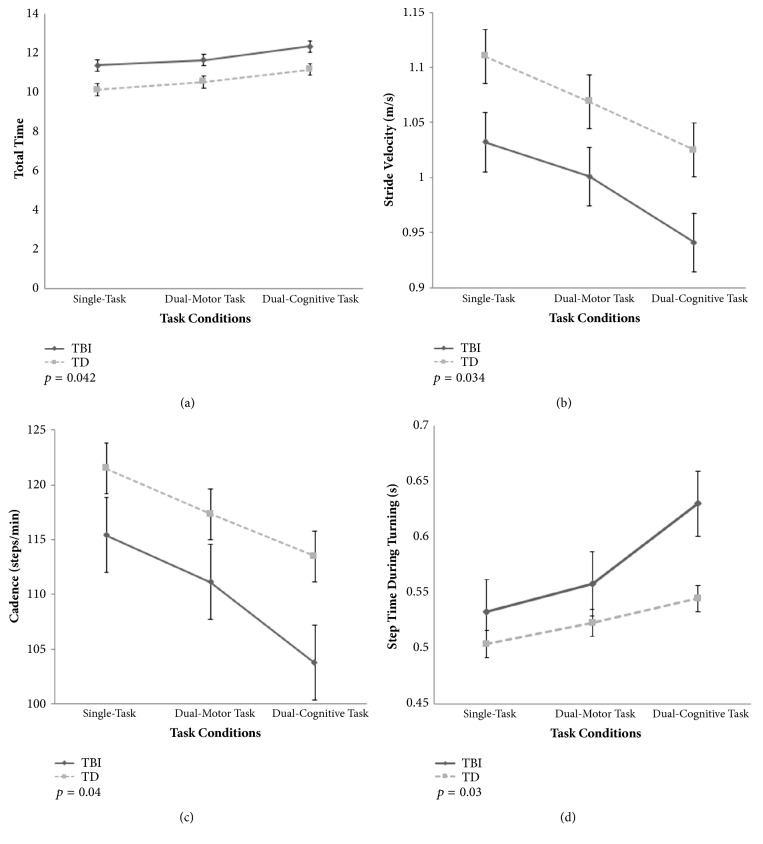
Comparison of gait parameters between dual-task conditions: (a) total time taken to complete the modified timed up and go test; (b) stride velocity of the straight walking phase; (c) cadence of the straight walking phase; (d) step time during turning. TBI, traumatic brain injury; TD, typically developing.

Overall, children with TBI required a longer time than TD children (see Figure 2(a)) to complete the modified TUG test (group effect, p = 0.042). The dual-task conditions significantly increased the time to complete the modified TUG test (condition effect, p = 0.001), and the effect was similar for both groups (condition × group interaction, p = 0.82). Post hoc comparisons indicated that the dual-cognitive task condition led to a significant increase in the time to complete the modified TUG test compared to the single-task and dual-motor task conditions (p = 0.001 for both), with a significant difference between the single-task and dual-motor task conditions (p = 0.003).

### 3.3. Stride Velocity

Children with TBI had a significantly slower stride velocity (see Figure 2(b)) than TD children (group effect, p = 0.034). The dual-task conditions significantly decreased the stride velocity (condition effect, p = 0.001), and the effect was similar for both groups (condition × group interaction, p = 0.61). Post hoc comparisons indicated that the dual-cognitive task condition led to a significant decrease in the stride velocity compared to the single-task and dual-motor task conditions for both groups. Of particular importance, the stride velocity measured under dual-cognitive task conditions in children with TBI was the lowest (0.94 ± 0.15 m/s) among all conditions (single-task = 1.03 ± 0.12 m/s; dual-motor task = 1.00 ± 0.11 m/s).

### 3.4. Stride Length

No significant difference in stride length was found for both groups (group effect, p = 0.85). In addition, the dual-task conditions had no significant effect on stride length (condition effect, p = 0.13), and no significant interaction was found for both groups (condition × group interaction, p = 0.37).

### 3.5. Cadence

A decrease in gait speed can be related to a decrease in cadence and stride length. In this study, children with TBI showed a lower cadence (see Figure 2(c)) than TD children (group effect, p = 0.04). The dual-task conditions significantly decreased the stepping rate (condition effect, p = 0.001), and the effect was similar for both groups (condition × group interaction, p = 0.08). Post hoc comparisons indicated a significant difference in cadence among any pair (single-task vs. dual-motor task, p = 0.001; single-task vs. dual-cognitive task, p = 0.001; dual-motor task vs. dual-cognitive task, p = 0.001), and the effect was not significant for both groups (condition × group interaction, p = 0.08).

### 3.6. Gait Cycle Time

In contrast to cadence, no significant difference in gait cycle time was found between groups (group effect, p = 0.1). However, the dual-task conditions significantly increased the gait cycle time (condition effect, p = 0.001), and the effect was similar for both groups (condition × group interaction, p = 0.29). In addition, post hoc comparisons indicated that both the dual-motor (p = 0.001) and dual-cognitive (p = 0.001) task conditions led to a longer gait cycle time than the single-task condition.

### 3.7. Double Support

No significant difference in the percentage of double support was found for both groups (group effect, p = 0.09). However, the dual-task conditions led to a significant increase in the percentage of double support (condition effect, p = 0.001), and the effect was similar for both groups (condition × group interaction, p = 0.48). Post hoc comparisons indicated that the dual-task conditions (p = 0.001 for both) led to a greater percentage of double support than the single-task condition.

### 3.8. Stance and Swing Phase

Overall, no significant difference in stance and swing phases was found for both groups (group effect, p = 0.16 for both). However, the dual-task conditions led to a significant increase in stance phase and a decrease in swing phase (condition effect, p = 0.001 for both), and no significant interaction was found for both groups (condition × group interaction, p = 0.63 and p = 0.51, respectively). Post hoc comparisons indicated that, for the stance phase, the dual-cognitive task condition (p = 0.001) required a longer stance phase than the dual-motor and single-task conditions (p = 0.001, for both), whereas, for the swing phase, the dual-cognitive task condition (p = 0.001) led to a shorter swing phase than the dual-motor task and single-task conditions (p = 0.001, for both).

### 3.9. Turning Duration

The dual-task conditions had a significant effect on the time taken to complete the 180° turning (condition effect, p = 0.001), and the effect was similar for both groups (condition × group interaction, p = 0.44). Post hoc comparisons indicated that the dual-cognitive and dual-motor task conditions led to a longer time taken than the single-task condition (p = 0.001 and p = 0.004, respectively), with a significant difference between the dual-motor and dual-cognitive task conditions (p = 0.009). However, no significant difference was found between groups (group effect, p = 0.08).

### 3.10. Step Time during Turning

The average step time during turning was also affected by the dual-task conditions, which was confirmed by a significant condition effect (p = 0.001), and no significant interaction was found for both groups (condition × group interaction, p = 0.11). Post hoc comparisons indicated that both dual-task conditions (motor and cognitive) led to a longer time taken than the single-task condition (p = 0.001 for both). However, no significant difference was found between dual-motor and dual-cognitive task (p = 0.02). In addition, children with TBI took a longer step time during turning (see Figure 2(d)) than TD children (group effect, p = 0.03).

### 3.11. Step Time before Turning

Children with TBI did not differ from TD children with respect to the step time before turning (group effect, p = 0.22). However, the dual-task conditions led to a significant increase in the step time before turning (condition effect, p = 0.001), and no significant interaction was found for both groups (condition × group interaction, p = 0.12). Post hoc comparisons indicated that the dual-cognitive task condition led to the longest time taken among all conditions (single-task, p = 0.001; dual-motor task, p = 0.009), whereas the difference between single-task and dual-motor task conditions was not significant (p = 0.17).

## 4. Discussion

This study focused on comparing gait performance between children with TBI and TD children under dual-task conditions during the modified TUG test. Three significant findings were noted. First, gait performance deteriorated under dual-task (dual-motor and dual-cognitive task) conditions during the straight walking and turning phases of the modified TUG test in both groups. Second, children with TBI required a longer time to complete the modified TUG test under dual-task conditions than TD children. Third, step time during turning was longer in children with TBI under single-task and dual-task conditions than in TD children.

We found that all children with TBI and TD children showed a deterioration in gait performance when more than two tasks were simultaneously executed. However, the deterioration was more marked in children with TBI, especially under dual-cognitive task condition. This is possibly due to impairment in attention, executive function, and information processing combined with the greater demand for controlled cognitive processing [21]. Difficulties in performing more than two tasks have been explained by neuropsychological theories, including the bottleneck theory, capacity-sharing theory, and multiple resource theory. In fact, the result of this study reflects the capacity-sharing theory. According to this theory, task performance would lead to worsening of at least one of the tasks because of the limited-capacity attentional resources [22]. In this study, participants were asked to focus on the performance of the secondary tasks (holding a tray and counting backwards without committing error) instead of the walking and turning tasks. As a result, participants showed a deterioration in gait performance when more than two tasks were simultaneously executed.

Previous studies reported that adults with TBI walked more slowly with shorter steps [23]. Further, a few studies showed that children with TBI walked with slower gait velocity, shorter step length, and longer step time than healthy controls [2, 3]. The study by Katz et al. [6] showed that the concurrent performance of cognitive tasks caused a significant deterioration in gait velocity, step length, step time, and step length variability in children with TBI compared to that in TD children, and a similar recent study demonstrated that healthy children had decreased gait velocity and cadence under dual-task conditions compared to that under single-task condition [24]. In this study, children with TBI required a longer time to complete the modified TUG test under both dual-motor and dual-cognitive compared to single task because of the reduction in gait speed during the straight walking and turning phases. This could be due to greater attentional resources required, when compared to the single task. This greater deterioration in children with TBI may be explained by balance impairment presented in this population as depicted by the results of PBS. The balance impairment in children with TBI may have caused the dual tasks (motor and cognitive) to be more complex as it requires them to move and control more body segments while performing the tasks simultaneously.

We observed that, instead of increasing their number of steps, children with TBI took a longer step time during the turning phase. The reduction in gait speed may reflect adaptations needed to increase stability and prevent falls during dynamic control of walking. The dual-cognitive task condition seems to be more challenging for them than the dual-motor task and single-task conditions. This may be related to the stepping strategy used during the modified TUG test.

As expected, there was a difference in effect between the dual-cognitive and dual-motor task conditions. The dual-cognitive task condition seems to be more demanding than the dual-motor task condition, with the deterioration in gait performance being greater under the dual-cognitive task condition than under the dual-motor task condition. This is possibly because holding a tray would be less demanding for a child with intact upper extremity function. However, if a child has limited hand function, holding a tray can be just as demanding (or even more) than counting. Therefore, the higher the level of difficulty of a secondary task, the more impaired the gait performance. Furthermore, impairments in attentional and information processing tasks are associated with TBI; depending on the part of the cerebral cortex region affected by the injury and its level of maturity, differential outcome may be evident for the attentional areas examined [25].

This study had several limitations. First, the number of participants in this study was small, which limited the generalizability of the results. Second, with respect to the level of difficulty, the secondary motor tasks used may require less effort for those children with TBI who do not have upper limb motor impairments, and if possible, in a future study, more complex and difficult motor and cognitive tasks can be explored. Third, it is possible that the children had learned to adapt during the repeated trial even though the tasks were in randomized order. Fourth, we did not record the number of failed trials of the tasks for each group which leads to inability to assess comparing cognitive demand between groups.

## 5. Conclusions

In summary, safe walking and turning are important functional daily living tasks. The results of this study indicated that children with TBI are more susceptible to dual-task interference during walking and turning especially under dual-cognitive task. Therefore, it is recommended that attentional aspects of cognitive rehabilitation be incorporated into gait training for children after TBI.

## Figures and Tables

**Table 1:** Demographic information of children with TBI and TD children.

	TBI (n = 14)	TD (n = 21)	p-value
Age (year)	11.6 ± 2	11.4 ± 2.3	0.85
Body weight (kg)	40.4 ± 12.5	41.2 ± 17.1	0.89
Height (cm)	141 ± 17	142 ± 15	0.88
PBS score (max 56)	52.1 ± 5.8	55.5 ± 0.7	0.034^*∗*^
GCS score	9 ± 2		
Time away from event (year)	2.4 ± 1.5		

## Data Availability

The data used to support the findings of this study are available from the corresponding author based on reasonable request.

## References

[1] Dewan M. C., Mummareddy N., Wellons J. C., Bonfield C. M. (2016). Epidemiology of Global Pediatric Traumatic Brain Injury: Qualitative Review. *World Neurosurgery*.

[2] Katz-Leurer M., Rotem H., Keren O., Meyer S. (2009). The relationship between step variability, muscle strength and functional walking performance in children with post-traumatic brain injury. *Gait & Posture*.

[3] Katz-Leurer M., Rotem H., Lewitus H., Keren O., Meyer S. (2008). Relationship between balance abilities and gait characteristics in children with post-traumatic brain injury. *Brain Injury*.

[4] Niechwiej-Szwedo E., Inness E. L., Howe J. A., Jaglal S., McIlroy W. E., Verrier M. C. (2007). Changes in gait variability during different challenges to mobility in patients with traumatic brain injury. *Gait & Posture*.

[5] Woollacott M., Shumway-Cook A. (2002). Attention and the control of posture and gait: a review of an emerging area of research. *Gait & Posture*.

[6] Katz-Leurer M., Rotem H., Keren O., Meyer S. (2011). Effect of concurrent cognitive tasks on gait features among children post-severe traumatic brain injury and typically-developed controls. *Brain Injury*.

[7] Patla A. E. (2001). Mobility in complex environments: Implications for Clinical Assessment and Rehabilitation. *Neurology Report*.

[8] Haggard P., Cockburn J. (1998). Concurrent performance of cognitive and motor tasks in neurological rehabilitation. *Neuropsychological Rehabilitation*.

[9] Lord S., Rochester L. (2007). Walking in the real world: concepts related to functional gait. *NZ Journal of Physiotherapy*.

[10] Pashler H. (1994). Dual-task interference in simple tasks: data and theory. *Psychological Bulletin*.

[11] Katz-Leurer M., Rotem H., Lewitus H., Keren O., Meyer S. (2008). Functional balance tests for children with traumatic brain injury: Within-session reliability. *Pediatric Physical Therapy*.

[12] Williams E. N., Carroll S. G., Reddihough D. S., Phillips B. A., Galea M. P. (2005). Investigation of the Timed 'Up & Go' Test in children. *Developmental Medicine & Child Neurology*.

[13] Nicolini-Panisson R. D., Donadio M. V. F. (2014). Normative values for the Timed 'Up and Go' test in children and adolescents and validation for individuals with Down syndrome. *Developmental Medicine & Child Neurology*.

[14] Catena R. D., Van Donkelaar P., Chou L.-S. (2007). Cognitive task effects on gait stability following concussion. *Experimental Brain Research*.

[15] Manaf H., Justine M., Goh H.-T. (2015). Effects of attentional loadings on gait performance before turning in stroke survivors. *Physical Medicine and Rehabilitation*.

[16] Manaf H., Justine M., Ting G., Latiff L. (2014). Comparison of gait parameters across three attentional loading conditions during timed up and go test in stroke survivors. *Topics in Stroke Rehabilitation*.

[17] Mancini M., King L., Salarian A., Holmstrom L., Mcnames J., Horak F. B. (2012). Mobility lab to assess balance and gait with synchronized body-worn sensors. *Journal Bioengineer & Biomedical*.

[18] Zampieri C., Salarian A., Carlson-Kuhta P., Aminian K., Nutt J. G., Horak F. B. (2010). The instrumented timed up and go test: Potential outcome measure for disease modifying therapies in Parkinson's disease. *Journal of Neurology, Neurosurgery & Psychiatry*.

[19] Mancini M., Chiari L., Holmstrom L., Salarian A., Horak F. B. (2016). Validity and reliability of an IMU-based method to detect APAs prior to gait initiation. *Gait & Posture*.

[20] Duarte N. D. A. C., Grecco L. A. C., Franco R. C., Zanon N., Oliveira C. S. (2014). Correlation between pediatric balance scale and functional test in children with cerebral palsy. *Journal of Physical Therapy Science*.

[21] McCulloch K. L., Buxton E., Hackney J., Lowers S. (2010). Balance, attention, and dual-task performance during walking after brain injury: associations with falls history. *The Journal of Head Trauma Rehabilitation*.

[22] Yogev-Seligmann G., Hausdorff J. M., Giladi N. (2008). The role of executive function and attention in gait. *Movement Disorders*.

[23] Williams G., Galna B., Morris M. E., Olver J. (2010). Spatiotemporal deficits and kinematic classification of gait following a traumatic brain injury: A systematic review. *The Journal of Head Trauma Rehabilitation*.

[24] Beurskens R., Muehlbauer T., Granacher U. (2015). Association of dual-task walking performance and leg muscle quality in healthy children. *BMC Pediatrics*.

[25] Catroppa C., Anderson V., Godfrey C., Rosenfeld J. V. (2011). Attentional skills 10 years post-paediatric traumatic brain injury (TBI). *Brain Injury*.

